# Performance of 5 Prominent Large Language Models in Surgical Knowledge Evaluation: A Comparative Analysis

**DOI:** 10.1016/j.mcpdig.2024.05.022

**Published:** 2024-06-05

**Authors:** Adam M. Ostrovsky, Joshua R. Chen, Vishal N. Shah, Babak Abai

**Affiliations:** Department of Surgery, Thomas Jefferson University, Philadelphia, PA

Artificial intelligence (AI)–based large language models (LLMs) have emerged as a promising avenue for revolutionizing medical education. For its ability to process complex queries and provide tailored responses, dialog-based LLM chat generative pretrained transformer (ChatGPT) received widespread acclaim on release, boasting over 1 million users within days.^1^ Since then, alternatives including free options like Google Gemini and Bing CoPilot, paid options such as ChatGPT 4.0, and specialized platforms for medical professionals like Health Insurance Portability and Accountability Act compliant DoximityGPT, have emerged. Previous studies have found that LLMs have been able to answer surgical multiple-choice medical student-level and resident-level medical questions at or near the average human level.^2^^,^^3^ Although promising, they are underevaluated, particularly regarding reliability. This study investigated the accuracy and reliability of these LLMs head-to-head in answering multiple-choice surgery clerkship examination questions to elucidate their potential for enhancing surgical education.

### Methods

We reviewed 150 multiple-choice questions across 3 National Board of Medical Examiners (NBME) surgery clerkship self-assessment examinations (Form A, Form B, and Form C) available for purchase for medical students, residents, and practicing physicians on the NBME website.^4^ National Board of Medical Examiners subject examinations are typically used by medical schools to assess third-year medical students at the end of their respective clerkships. Each examination contains 110 questions delivered over a timespan of approximately 3 hours, with the average score for each examination differing greatly across each specialty (neurology, family medicine, internal medicine, obstetrics and gynecology, pediatrics, psychiatry, and surgery).^5^ Each question was individually queried using 5 LLMs (ChatGPT 3.5, ChatGPT 4.0, DoximityGPT, Google Gemini, and Bing CoPilot) in March 2024, with data reset after each question to avoid bias. Each question was input with no prompting strategy, and 3 trials were performed for each form on each LLM. No questions were excluded before analysis. A question was marked “always correct” when an LLM could answer it correctly across all 3 trials, and “always wrong” when an LLM provided incorrect answers across all 3 trials. Questions answered correctly by an LLM on either 1 or 2 of 3 trials was marked “sometimes correct,” and responses with no direct answer were marked as incorrect. Data were analyzed using descriptive statistics. Test-retest reliability was calculated for the 3 trials using Fleiss κ statistic (R package, “irr”; R V4.3.3) with significance set at *P*<.05, and data were visualized using GraphPad Prism (version 10.2.2).

### Results

Mean accuracy for the 3 trials of each test form run on each LLM was highest for DoximityGPT (94.9%±3.0%), followed by ChatGPT 4.0 (91.3%±1.6%), ChatGPT 3.5 (72.4%±4.2%), Gemini (64.2%±4.9%), and CoPilot (62.4%±5.1%) (Figure). Test-retest reliability was highest for DoximityGPT (κ=0.94; *P*<.01), followed by ChatGPT 4.0 (κ=0.90; *P*<.001), Gemini (κ=0.81; *P*<.001), ChatGPT 3.5 (κ=0.78; *P*<.001), and CoPilot (κ=0.59; *P*<.001). DoximityGPT was most often always right across trials (91.3%), and CoPilot was least often always right (40%) (Table). A more granular breakdown of how each LLM performed overall on each subcategory of questions (eg, central nervous system, musculoskeletal, and gastrointestinal) is available in Supplemental Table (available online at https://www.mcpdigitalhealth.org/).

**Figure:**
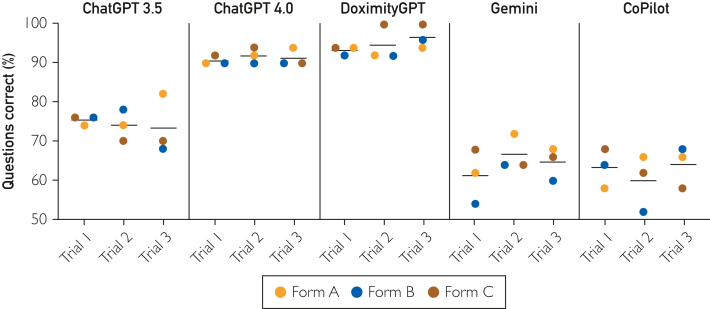
Comparison of performance of 5 LLMs on 3 different NBME surgery clerkship self-assessment examination forms, with 3 trials performed for each examination. The black bars demonstrate the mean scores attained by each LLM across the 3 trials conducted for each form. ChatGPT, chat generative pretrained transformer; LLM, large language model; NBME, National Board of Medical Examiners.

**Table:** Large Language Model Trial Results

	ChatGPT 3.5	ChatGPT 4.0	DoximityGPT	Gemini	CoPilot
Form A					
Always right (%)	70	88	92	56	40
Sometimes right (%)	16	8	4	22	42
Always wrong (%)	14	4	4	22	18
Trial 1 (%)	74	90	94	62	58
Trial 2 (%)	76	90	92	54	64
Trial 3 (%)	76	92	94	68	68
Mean ± SD (%)	75.33±0.94	90.67±0.94	93.33±0.94	61.33±5.70	63.33±4.10
Form B					
Always right (%)	62	84	88	50	42
Sometimes right (%)	24	14	12	20	36
Always wrong (%)	14	2	0	30	22
Trial 1 (%)	74	92	92	72	66
Trial 2 (%)	78	90	92	64	52
Trial 3 (%)	70	94	100	64	62
Mean ± SD (%)	74.00±3.30	92.00±1.60	94.67±3.80	66.67±3.80	60.00±5.90
Form C					
Always right (%)	60	86	94	60	38
Sometimes right (%)	26	12	6	12	46
Always wrong (%)	14	2	0	28	16
Trial 1 (%)	82	94	94	68	66
Trial 2 (%)	68	90	96	60	68
Trial 3 (%)	70	90	100	66	58
Mean ± SD (%)	73.33±6.20	91.33±1.90	96.67±2.50	64.67±3.40	64.00±4.30

Abbreviation: ChatGPT, chat generative pretrained transformer; SD, standard deviation.

### Discussion and Conclusion

This study contributes to the growing body of research on the application of LLMs in medicine by shedding light on the accuracy and reliability of these models in the context of answering medical questions. Among the LLMs tested, DoximityGPT and ChatGPT 4.0 exhibited the highest levels of accuracy and reliability. Notably, DoximityGPT was the only LLM to achieve 100% accuracy in any trial. This is a substantial finding, given that accuracy in medical knowledge dissemination is crucial for the safe and effective practice of medicine. Previous studies have found the ability of LLMs to pass standardized medical examinations, indicating their potential as learning tools. However, these studies have been limited in scope, focusing only on specific models such as ChatGPT or outdated chatbots.^6^, ^7^, ^8^ Our study compares a wider array of available LLMs, providing a more comprehensive picture of the current landscape.

One of our key findings is that all free, universally available LLMs were both less accurate and less reliable compared with paid and provider-specific LLMs. This suggests that although free LLMs may offer widespread accessibility, their use may come at the cost of accuracy and reliability. Medical professionals and students may need to consider these trade-offs when selecting an LLM for their learning needs. Interestingly, our study also found that LLMs may provide differing responses to the same questions across multiple trials. This variability in AI-generated responses, even to identical queries with choices, is noteworthy. Although individuals typically pose questions only once, our findings underscore the importance of interpreting responses with caution, particularly when there are no objective measures to validate their accuracy. When compared with the nationwide performance of new clinical medical students on the NBME surgery clerkship examination,^5^ LLM performance across all forms averaged in the 12th percentile for CoPilot, 17th percentile for Gemini, 55th percentile for ChatGPT 3.5, 100th percentile for ChatGPT 4.0, and 100th percentile for DoximityGPT. This also suggests that the use of LLMs in medical education should be supplemented with other sources of information and validation.

This study is not without limitations. The narrow scope of questions used may limit the generalizability of our findings to other medical domains or question types. In addition, the observed variability in AI-generated responses across multiple trials raises questions about the reliability and consistency of these models. These limitations highlight the need for further investigation into factors influencing response variations and the potential biases within the training data. Moreover, our study did not delve into the underlying mechanisms driving differences in AI performance. Understanding these mechanisms is crucial for the improvement and optimization of LLMs. Future research should explore potential biases within the training data because these could considerably impact the accuracy and reliability of AI-generated responses.

In the realm of AI, patient privacy is a critical concern.^9^ The use of LLMs in health care potentially involves the processing and storage of sensitive patient information, raising privacy issues. Some LLMs like DoximityGPT are taking steps to address these concerns by implementing data encryption to be Health Insurance Portability and Accountability Act compliant. This brings them one step closer to meeting the demands for possible future clinical applications, including diagnostic support and patient risk stratification and triage.^10^ Although LLMs hold great promise, their use should be guided by careful consideration of their accuracy, reliability, and adherence to privacy standards. As the field of AI continues to evolve, ongoing research and evaluation are essential to ensure that these models are used effectively and responsibly.

## Potential Competing Interests

The authors report no competing interests.
