# Supplementary material for: Performance of 5 Prominent Large Language Models in Surgical Knowledge Evaluation: A Comparative Analysis
Source: Mayo Clin Proc Digit Health. 2024 Jun 5;2(3):348–50. doi: 10.1016/j.mcpdig.2024.05.022 (PMC11975981; doi:10.1016/j.mcpdig.2024.05.022)
Supplement: Supplement Table 1 [file mmc1.pdf]

Supplement Table 1: Large Language Model Trial Results By Question Category, Across Three Trials.

Form A						
**Question Category**	**Total Questions Across Three Trials**	**Total Questions Correct**				
		ChatGPT 3.5	ChatGPT 4.0	DoximityGPT	Gemini	CoPilot
Immune	3	1 (33.3%)	3 (100%)	3 (100%)	2 (66.7%)	0 (0%)
Blood	9	9 (100%)	9 (100%)	9 (100%)	9 (100%)	7 (77.8%)
CNS	9	9 (100%)	9 (100%)	9 (100%)	7 (77.8%)	6 (66.7%)
Skin	3	0 (0%)	3 (100%)	3 (100%)	0 (0%)	0 (0%)
MSK	6	1 (16.7%)	6 (100%)	6 (100%)	5 (83.3%)	5 (83.3%)
Cardio	9	6 (66.7%)	9 (100%)	9 (100%)	6 (66.7%)	5 (55.6%)
Resp	15	13 (86.7%)	15 (100%)	13 (86.7%)	12 (80.0%)	9 (60.0%)
GI	45	33 (73.3%)	38 (84.4%)	40 (88.9%)	19 (42.2%)	29 (64.4%)
Renal	9	4 (44.4%)	5 (55.6%)	6 (66.7%)	8 (88.9%)	6 (66.7%)
Repro	15	12 (80.0%)	15 (100%)	15 (100%)	9 (60.0%)	7 (46.7%)
Endo	12	12 (100%)	11 (91.7%)	12 (100%)	9 (75.0%)	11 (91.7%)
Multi	12	12 (100%)	12 (100%)	12 (100%)	12 (100%)	10 (83.3%)
Soc. Sci.	3	3 (100%)	3 (100%)	3 (100%)	3 (100%)	0 (0%)
**Form B**						
**Question Category**	**Total Questions Across Three Trials**	ChatGPT 3.5	ChatGPT 4.0	DoximityGPT	Gemini	CoPilot
Immune	3	3 (100%)	3 (100%)	3 (100%)	3 (100%)	3 (100%)
Blood	9	6 (66.7%)	9 (100%)	9 (100%)	6 (66.7%)	6 (66.7%)
CNS	12	11 (91.7%)	12 (100%)	12 (100%)	6 (50.0%)	10 (83.3%)
Skin	6	6 (100%)	6 (100%)	6 (100%)	1 (16.7%)	4 (66.7%)
MSK	9	6 (66.7%)	9 (100%)	9 (100%)	1 (11.1%)	7 (77.8%)
Cardio	21	17 (81.0%)	21 (100%)	21 (100%)	21 (100%)	14 (66.7%)
Resp	15	9 (60.0%)	12 (80.0%)	13 (86.7%)	8 (53.3%)	8 (53.3%)
GI	33	22 (66.7%)	27 (81.8%)	32 (97.0%)	16 (48.5%)	14 (42.4%)
Renal	9	8 (88.9%)	9 (100%)	9 (100%)	6 (66.7%)	8 (88.9%)
Repro	12	8 (66.7%)	12 (100%)	12 (100%)	6 (50.0%)	6 (50.0%)
Endo	9	5 (55.6%)	6 (66.7%)	6 (66.7%)	6 (66.7%)	4 (44.4%)
Multi	9	7 (77.8%)	6 (66.7%)	5 (55.6%)	7 (77.8%)	5 (55.6%)
Soc. Sci.	3	3 (100%)	3 (100%)	3 (100%)	2 (66.7%)	3 (100%)

**Form C**						
**Question Category**	**Total Questions Across Three Trials**	ChatGPT 3.5	ChatGPT 4.0	DoximityGPT	Gemini	CoPilot
Immune	0					
Blood	9	7 (77.8%)	9 (100%)	9 (100%)	3 (33.3%)	7 (77.8%)
CNS	6	4 (66.7%)	6 (100%)	6 (100%)	6 (100%)	5 (83.3%)
Skin	9	7 (77.8%)	8 (88.9%)	9 (100%)	6 (66.7%)	3 (33.3%)
MSK	6	6 (100%)	6 (100%)	6 (100%)	6 (100%)	5 (83.3%)
Cardio	15	10 (66.7%)	12 (80.0%)	15 (100%)	9 (60.0%)	11 (73.3%)
Resp	15	8 (53.3%)	14 (93.3%)	14 (93.3%)	6 (40.0%)	9 (60.0%)
GI	48	34 (70.8%)	45 (93.8%)	46 (95.8%)	31 (64.6%)	23 (47.9%)
Renal	6	6 (100%)	6 (100%)	6 (100%)	4 (66.7%)	6 (100%)
Repro	12	8 (66.7%)	12 (100%)	12 (100%)	9 (75.0%)	9 (75.0%)
Endo	9	9 (100%)	9 (100%)	9 (100%)	6 (66.7%)	7 (77.8%)
Multi	9	6 (66.7%)	7 (77.8%)	9 (100%)	7 (77.8%)	7 (77.8%)
Soc. Sci.	6	3 (50.0%)	4 (66.7%)	6 (100%)	6 (100%)	2 (33.3%)
